# Re: CaRMS at 50

**DOI:** 10.36834/cmej.71154

**Published:** 2020-12-07

**Authors:** Malcolm MacFarlane

**Affiliations:** 1 Society Of Canadians Studying Medicine Abroad, British Columbia, Canada

In April 2020, CMEJ published an article “CaRMS at 50: Making the match for medical education” that highlighted the application and matching system over the past fifty years.^1^ CaRMS at 50 certainly has much to celebrate. However, for an organization that was built around “fairness and equity, which remain core values today,”^1^ the paper fails to address the unfair and inequitable treatment of international medical graduates (IMGs) in the CaRMS match.

The authors refer to 1,725 IMGs in the 2019 match, and to “significant growth” in IMG matches exemplified by over 300 matches annually. The article makes no effort to address the contentious policy of streaming of IMGs to limited residency positions.

The paper highlights the issue of unmatched Canadian Medical Graduates (CMGs) while glossing over the issue of unmatched IMGs. In the 2020 match, there were 3072 positions for about 3000 CMGs resulting in a 97% match rate. In contrast, there were only 325 IMG positions available to over 1800 IMG applicants resulting in about an 18% match rate.

To apply to CaRMS, IMGs must be Canadian Citizens or permanent residents, just like CMGs, and should be subject to equal treatment. Instead, in keeping with AFMC’s 2006 resolution that all CMGs should receive residency positions, CMGs are privileged and IMGs are marginalized.

This inequity has been raised in the press and is the subject of a human rights challenge in BC. Some studies have examined discrimination faced by IMGs,^2^ but to my knowledge there is little in the academic literature that directly addresses marginalization of IMGs in the CaRMS match process. These inequities remain unacknowledged, and the authors make no attempt to raise this important issue.

While CaRMS eligibility criteria are set by the provincial faculties of medicine and Ministries of Health, eligibility criteria for IMGs in all provinces includes standardized assessments of competence such as the MCCQE1 and the NAC OSCE. IMG applicants have objectively demonstrated their competence.

If part of the purpose of a competitive match is to select the most competent future physicians for residency training, does CaRMS not have a responsibility to raise this issue when 1,400 competent IMGs who are Canadian citizens or permanent residents are unmatched due to an inequitable process? Should this not be a major concern for an organization that values fairness?

In the context of the Canadian Medical Association’s recently published Policy on Equity and Diversity in Medicine^3^ this avoidance of meaningful dialogue is severely disappointing. The CMA policy advocates “opening the conversation to include the voices and knowledge of those who have historically been under-represented and/or marginalized.”

Such conversations might transparently explore the public interest and merits of selecting the most competent residents vs. protecting the public’s investment in CMG undergraduate medical education. It might examine the societal cost of underutilization of unmatched IMGs when five million Canadians are without family physicians. It might explore whether current eligibility criteria are consistent with societal objectives of encouraging international labour mobility, or with established principles for recognition of international credentials such as the Lisbon Recognition Convention. Finally, it might explore ways to increase IMG representation as stakeholders in organizations that make decisions affecting their interests.

There is ample space for CaRMS to take a leadership role in speaking out about the inequities of the current match and, as the CMA policy advocates, “reducing the structural barriers faced by those who want to enter the medical profession.” IMGs in the CaRMS match are all Canadian citizens or permanent residents. Prime Minister Trudeau says, “a Canadian is a Canadian.” Maybe, but not for IMGs.
